# New Insights Into DNA Repair Revealed by NucS Endonucleases From Hyperthermophilic Archaea

**DOI:** 10.3389/fmicb.2020.01263

**Published:** 2020-07-02

**Authors:** Likui Zhang, Donghao Jiang, Mai Wu, Zhihui Yang, Philippe M. Oger

**Affiliations:** ^1^ College of Environmental Science and Engineering, Marine Science and Technology Institute, Yangzhou University, Yangzhou, China; ^2^ College of Plant Protection, Agricultural University of Hebei, Baoding, China; ^3^ Univ Lyon, INSA de Lyon, CNRS UMR 5240, Villeurbanne, France; ^4^ Guangling College, Yangzhou University, Yangzhou, China

**Keywords:** hyperthermophilic Archaea, NucS endonuclease, mismatch repair, nucleotide excision repair, deaminated DNA repair

## Abstract

Hyperthermophilic Archaea (HA) thrive in high temperature environments and their genome is facing severe stability challenge due to the increased DNA damage levels caused by high temperature. Surprisingly, HA display spontaneous mutation frequencies similar to mesophilic microorganisms, thereby indicating that the former must possess more efficient DNA repair systems than the latter to counteract the potentially enhanced mutation rates under the harsher environment. Although a few repair proteins or enzymes from HA have been biochemically and structurally characterized, the molecular mechanisms of DNA repair of HA remain largely unknown. Genomic analyses of HA revealed that they lack MutS/MutL homologues of the mismatch repair (MMR) pathway and the recognition proteins of the nucleotide excision repair (NER) pathway. Endonucleases play an essential role in DNA repair. NucS endonuclease, a novel endonuclease recently identified in some HA and bacteria, has been shown to act on branched, mismatched, and deaminated DNA, suggesting that this endonuclease is a multifunctional enzyme involved in NER, MMR, and deaminated base repair in a non-canonical manner. However, the catalytic mechanism and the physiological function of NucS endonucleases from HA need to be further clarified to determine how they participate in the different DNA repair pathways in cells from HA. In this review, we focus on recent advances in our understanding of the function of NucS endonucleases from HA in NER, MMR, and deaminated DNA repair, and propose directions for future studies of the NucS family of endonucleases.

## Introduction

Archaea compose the third domain of life, and the second domain of the prokaryotes. As such, they have a single-cell ultrastructure with no cell nucleus, similar to bacteria. However, Archaea share high similarities to eukaryotic cells for many central cellular processes, such as DNA replication and RNA polymerization, suggesting that they are a simplified version of eukaryotes for several functions, which is coherent with the currently proposed origins of eukaryotes from an archaeal ancestor (Eme et al., 2017). Archaeal DNA replication, DNA repair, and DNA recombination (White and Allers, 2018) are also share extensive similarities to that of eukaryotes. Hyperthermophilic Archaea (HA) are the Archaea with an optimal growth temperature above 80°C (Stetter, 2013), where they live in high temperature environments such as high-pressure hydrothermal vents, volcanic vents, and hot springs. Since the isolation of the first hyperthermophilic crenarchaeon *Sulfolobus acidocaldarius* from Yellowstone Park, more than 90 species of HA have been identified, comprising most members of the Euryarchaea and the Crenarchaea (Stetter, 2013; Eme et al., 2017). Studying HA helps us to understand adaptation mechanisms to life in high temperature environments and develop thermostable enzyme resources, and also provides new insights into the origin and evolution of life.

Endogenous and exogenous factors are capable of causing DNA damage, following which the replication of the damaged DNA would lead to serious consequences for the cells, such as the accumulation of mutations and eventual cell death, if it is not repaired. Thus, efficient DNA repair is an essential cellular function to maintain genomic integrity. The high temperature environments in which HA thrive can further increase their genomic DNA damages, and thus increase drastically the challenge their genomes is facing (Grogan, 1998, 2015). Firstly, high temperature accelerates the deamination rates of bases in DNA (Lindahl and Nyberg, 1974), forming deaminated bases. Hypoxanthine, xanthine, and uracil are common deaminated bases, which are created by deamination of adenine, guanine, and cytosine, respectively. Secondly, high temperature also enhances the hydrolysis rates of DNA bases, potentially leading to elevated apurinic/apyrimidinic (AP) site levels in the cells of HA (Lindahl and Nyberg, 1972). For instance, the hyperthermophilic euryarchaeon *Pyrococcus abyssi* harbors a level of AP sites 10-fold higher than that of *Escherichia coli* (Palud et al., 2008). However, how HA maintain their genome integrity remains elusive (Grogan, 2000).

The molecular mechanisms of DNA repair, including nucleotide excision repair (NER), base excision repair (BER), mismatch repair (MMR), homologous recombination (HR) repair, and non-homologous end joining (NHEJ), have been extensively studied. These pathways are essentially conserved from prokaryotes to eukaryotes (Zatopek et al., 2018). As mentioned above, although they live in high temperature environments and experience higher mutagenic potential, HA harbor spontaneous mutation rates similar to those of *E. coli* (Jacobs and Grogan, 1997; Grogan et al., 2001). Thus, HA are expected to have developed more efficient repair systems than other organisms.

To date, the DNA repair pathways of HA have been receiving much attention because they remain to be fully understood. Genomic analyses of HA showed that they could encode a variety of proteins involved in the NER, BER, alkyl transfer, damage reversion, and TLS pathways (Stetter, 2013), but they lack MutS/MutL homologues of the conserved MMR pathway and the recognition proteins of the conserved NER pathway (Grogan, 2004). Although biochemical and structural data of several DNA repair proteins from HA have provided some insights into the catalytic mechanisms and repair pathways (Stetter, 2013), most of the DNA repair mechanisms of HA remain unclear. Endonucleases play an important role in DNA repair pathway since they can cleave the damaged DNA (Li et al., 2019). Recently, a novel endonuclease, NucS [nuclease specific for single-stranded DNA (ssDNA)], was identified and characterized in several HA and some bacteria. The biochemical, structural, and genetic analyses showed that NucS endonucleases from HA are multifunctional endonucleases that can cleave branched DNA, mismatched, and deaminated, potentially providing an alternative pathway for the removal of mismatches, deaminated bases, and helix-distorting DNA lesions from DNA in HA. The substrate specificity of NucS endonucleases from HA is summarized in Table 1.

**Table 1:** Substrate specificity of NucS endoncleases from hyperthermophilic Archaea (HA).

Organism	Substrate specificity	References
*Pyrococcus abyssi*	Branched and splayed DNA	(Ren et al., 2009)
*Thermococcus kodakarensis*	Mismatched DNA; I-containing dsDNA	(Ishino et al., 2016)
*Thermococcus gammatolerans*	U- and I-containing dsDNA; branched and splayed DNA; mismatched DNA	(Zhang et al., 2020)

## Archaeal NucS Endonucleases in Nucleotide Excision Repair

It is now well-known that NER is employed to repair helix-distorting DNA lesions, including UV-induced DNA damage (TT dimer and 6,4-photoproduct), intra-strand crosslinks, and bulky adducts (Grogan, 2015; White and Allers, 2018). NER proceeds in three steps: damage recognition, dsDNA opening by a helicase, and DNA cutting on both sides of the lesion by an endonuclease. Genomic analysis of HA have demonstrated that they possess homologues of only a fraction of the eukaryotic NER proteins, which comprise the XPF and XPG endonucleases, and the XPB and XPD helicases (Rouillon and White, 2011; Grogan, 2015; White and Allers, 2018) while they lack those for damage recognition proteins XPC and XPA (Grogan, 2015; White and Allers, 2018). Thus, it is widely admitted that HA do not possess a classic NER system as observed in other organisms and that their corresponding repair might involve other mechanisms.

Helix-distorting DNA damage can lead to stalled-fork DNA. To repair such stalled fork, HA could use a HR-mediated stalled-fork DNA repair pathway (Fujikane et al., 2010; Grogan, 2015). The stalled replication fork can be cleaved by a flap endonuclease that can act on branched DNA, and the corresponding lesion is then removed by double-stranded end processing. Genetic and biochemical data suggest that the 3'-flap endonuclease XPF/Hef (Hef represents euryarchaeal XPF) participates in an initial step of HR-mediated stalled-fork DNA repair (Nishino et al., 2005; Roberts and White, 2005). However, the 5'-flap endonuclease XPG/FEN-1 and other flap endonucleases do not seem to be involved in the helix-distorting DNA repair pathways of HA.

**Figure 1:**
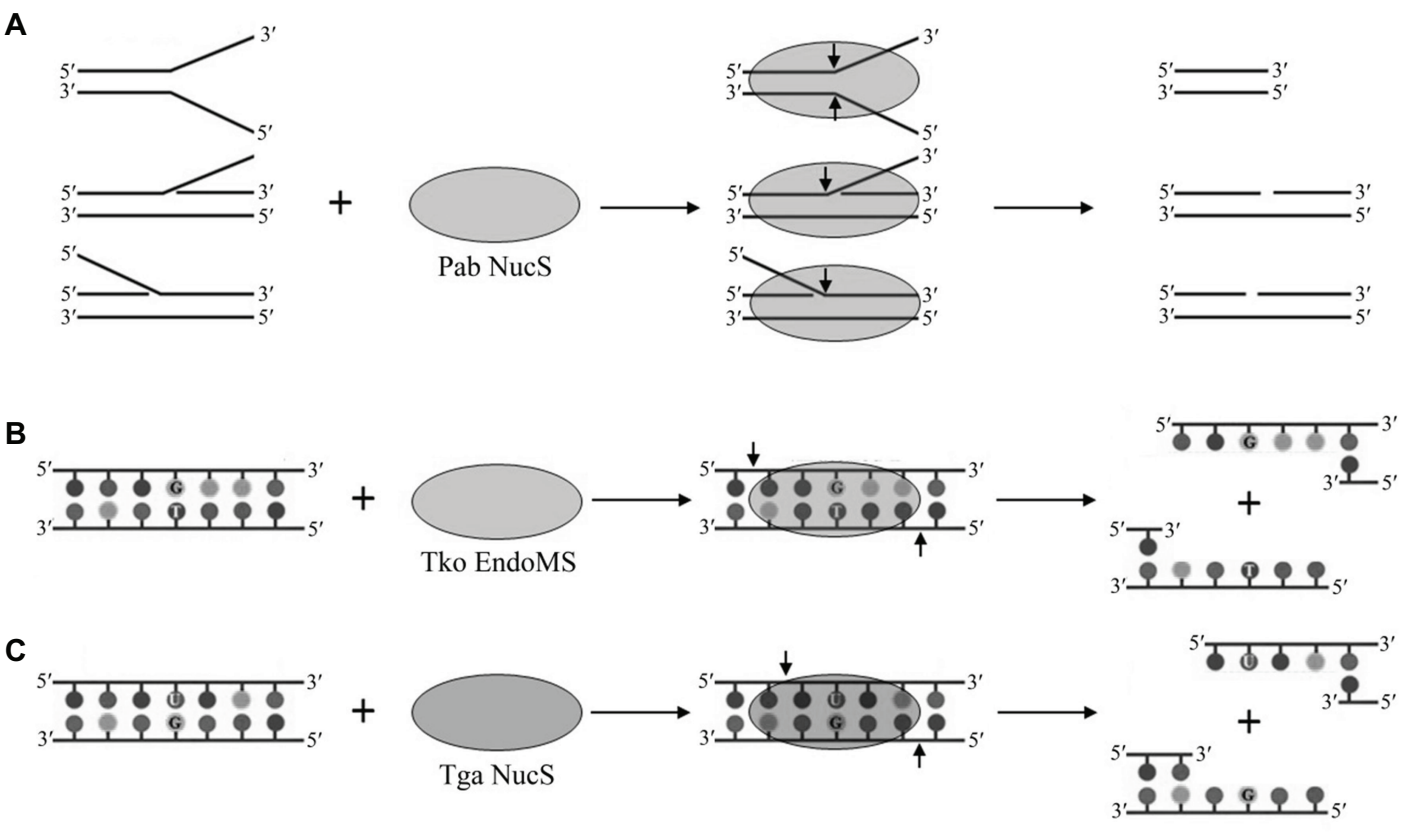
Cleavage of branched, mismatched, and deaminated DNA by archaeal NucS endonucleases. **(A)** Cleavage of branched DNA by Pab NucS. **(B)** Cleavage of mismatched DNA by Tko EndoMS. **(C)** Cleavage of deaminated DNA by Tga NucS.

HA genome sequences showed that they possess structure-specific endonucleases (Grogan, 2004), which might play a potential role in the recognition of helix-distorting DNA damage in their putative NER pathway, including the NucS endonuclease. NucS endonuclease from the hyperthermophilic euryarchaeon *P. abyssi* was first characterized *in vitro*, demonstrating that it can cleave the branched-structure DNA substrates that contain flapped and splayed DNA by removing the large ssDNA segments at the branch points (Figure 1A; Ren et al., 2009; Creze et al., 2011, 2012). The cleavage of flapped and splayed DNA substrates by Pab NucS suggests that this endonuclease is a potential archaeal NER endonuclease that can remove helix-distorting DNA damages (Rouillon and White, 2011). Furthermore, Rezgui et al. (2014) revealed that NucS endonuclease is a bipolar structure-specific endonuclease. *In vivo*, the engineered NucS-knockout strains of *S. acidocaldarius* are sensitive to DNA adducts, however, they did not show higher mutation rates (Suzuki and Kurosawa, 2019). Sac NucS does not seem to be involved in the DNA repair of UV-induced DNA damage, double strand break (DSB), or oxidative DNA damage, but this endonuclease seems essential for DNA repair of intra-strand crosslinks and works with XPF *via* the homologous recombination-mediated stalled-fork DNA repair for the removal of helix-distorting DNA lesions (Suzuki and Kurosawa, 2019). Combined with the cleavage of branched DNA substrates by Pab NucS, the role of Sac NucS *in vivo* in *Sulfolobus* cells suggests that the archaeal NucS endonuclease may be important in the NER pathway as a flap endonuclease.

## Archaeal NucS Endonucleases in Mismatch Repair

Most HA lack a complete mismatch repair machinery as the one conserved in bacteria and eukaryotes since they have no homologs of MutS and MutL (Grogan, 2004), the genes which are essential to recognize and remove mismatched bases. The search for an alternative MMR pathway in the hyperthermophilic euryarchaeon *Pyrococcus furiosus* leads to the isolation of a novel endonuclease, which was capable of cleaving mismatch-specific DNA in a non-canonical manner. This enzyme was named EndoMS, suggesting that this endonuclease is involved in DNA mismatch repair (Ishino et al., 2016). EndoMS proved to be identical to the NucS endonuclease previously characterized in *P. abyssi* (Ren et al., 2009). *Thermococcus kodakarensis* EndoMS (Tko EndoMs) is capable of cleaving a DNA double strand with a mismatched base, leaving a 5 base 5' overhang (Figure 1B; Ishino et al., 2016). Tko EndoMS displays a higher affinity for mismatched DNA than for branched or ssDNA. Furthermore, Tko EndoMS exhibits mismatched cleavage activity against G:T, G:G, T:T, T:C, and A:G mismatches, but cannot cleave C:C, A:C, or A:A mismatches *in vitro* (Ishino et al., 2016), which is consistent with the involvement of this enzyme in the excision of mismatched G or T. Structural data of Tko EndoMS suggest that the mismatched DNA substrate is wrapped by this endonuclease (Nakae et al., 2016), and two mismatched bases are flipped from the DNA substrate and cleaved by this endonuclease in a manner similar to type II restriction endonucleases (Mariko and Kosuke, 2016).

**Figure 2:**
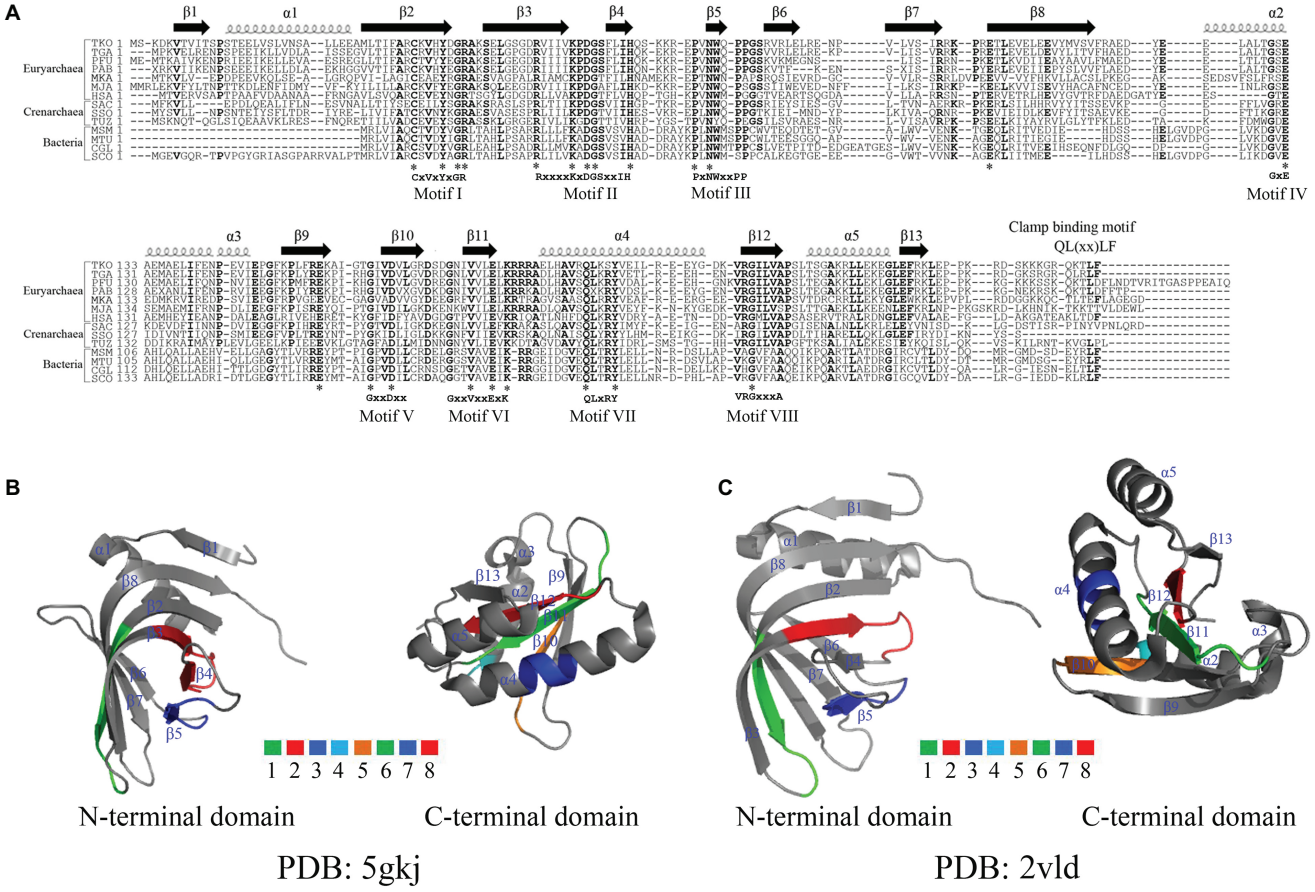
Alignment of amino acid residues and crystal structures of NucS endonucleases. **(A)** Alignment of amino acid residues of NucS endonucleases. Abbreviations used are as follows: TKO, *T. kodakarensis* (NCBI reference sequence: WP_011250849.1); TGA, *T. gammatolerans* (NCBI reference sequence: WP_015857754.1); PFU, *Pyrococcus furiosus* (NCBI reference sequence: WP_014835498.1); PAB, *Pyrococcus abyssi* (PDB: 2VLD_B); MKA, *Methanopyrus kandleri* (UniProtKB/Swiss-Prot: Q8TY00.1); MJA, *Methanocaldococcus jannaschii* (UniProtKB/Swiss-Prot: Q57678.1); HSA, *Halobacterium salinarum* (GenBank: QCC45644.1); SAC, *Sulfolobus acidocaldarius* (UniProtKB/Swiss-Prot: Q4JC60.1); SSO, *Sulfolobus solfataricus* (UniProtKB/Swiss-Prot: Q97WK8.1); TUZ, *Thermofilum uzonense* (NCBI reference sequence: WP_052884192.1); MSM, *Mycolicibacterium smegmatis* (NCBI reference sequence: WP_003896320.1); MTU, *Mycobacterium tuberculosis* (GenBank: SIP67590.1); CGL, *Corynebacterium glutamicum* (GenBank: QDQ24257.1); SCO, *Streptomyces coelicolor* (NCBI reference sequence: WP_003973608.1). The conserved amino acid residues are labeled in bold. ^*^Represents same amino acid residues in the selected organisms. **(B,C)** The crystal structures and conserved motifs of Tko EndoMS **(B)** and Pab NucS **(C)**. Tko EndoMS and Pab NucS are represented with α-helix and β-sheet, which are adapted from the references (Ren et al., 2009 and Nakae et al., 2016) by PyMOL. The conserved motifs in **(B)** and **(C)** are labeled with colors.

Besides HA and halophilic Archaea, a few bacteria that lack the MutS/MutL homologues also encode a NucS endonuclease (Figure 2A). Recent studies revealed that the deletion of the NucS endonuclease gene in *Mycobacterium smegmatis* leads to about 100-fold increased mutation rates, thereby creating a hypermutational phenotype (Castaneda-Garcia et al., 2017). Further mutational spectrum data demonstrated that the increased mutation rates are due to elevated levels of base conversion (A:T to G:C conversion or G:C to A:T conversion), which are the mutations created by a typical MMR defect (Castaneda-Garcia et al., 2017). Similar effects were observed in *Corynebacterium glutamicum* and *Streptomyces coelicolor* (Ishino et al., 2018; Takemoto et al., 2018). The *C. glutamicum* EndoMS was further confirmed as the mismatch-specific endonuclease and its function was fully dependent on its physical interaction with the sliding clamp. *In vivo*, a combination of the *C. glutamicum* EndoMS gene disruption and a mutation in the *dnaE* gene can lead to the increased mutation rates, thereby confirming the role of EndoMS in replication error correction (Ishino et al., 2018). *In vitro*, the *C. glutamicum* EndoMS can specifically cleave G/T, G/G, and T/T mismatches (Ishino et al., 2018), consistent with the mutation spectrum observed by genome-wide analyses. Thus, the bacterial NucS endonucleases resemble their archaeal homologues, and are capable of acting on mismatched bases in DNA to potentially complete the MMR DNA repair pathway.

## Archaeal NucS Endonucleases in Deaminated Base Repair

As mentioned above, high temperature leads to the accumulation of increased levels of uracil, hypoxanthine, or AP sites in genomic DNA. BER present in all organisms is a classical pathway for repairing the above-mentioned damaged bases (Grasso and Tell, 2014). Besides BER, the alternative excision repair (AER) pathway was identified as an alternative route to repair uracil, hypoxanthine, or AP sites. Generally, the AER pathway is initiated by an endonuclease that can cleave a phosphodiester bond of the DNA in the vicinity of the damaged base, thus creating a nick (Yasui, 2013). The endonuclease V (EndoV) was the first endonuclease described to mediate the AER pathway, capable of recognizing and cleaving hypoxanthine-containing DNA with a cleavage site downstream of the damaged base of the second phosphodiester bond. The genomes of most HA encode a conserved EndoV suggesting that they can use the AER pathway for the repair of uracil, hypoxanthine, or AP sites (Liu et al., 2000; Kanugula et al., 2005; Kiyonari et al., 2014; Wang et al., 2018). The HA EndoVs can cleave hypoxanthine-containing DNA in a similar manner as other EndoVs.

Recently, another endonuclease, EndoQ was characterized from the hyperthermophilic euryarchaeon *P. furiosus* (Ishino et al., 2015). Similar to EndoVs, Pfu EndoQ is capable of cleaving the 5′-end DNA phosphodiester bond of uracil, hypoxanthine, or abasic sites forming a nick (Shiraishi et al., 2015). Further studies suggested that EndoQ and EndoV from *P. furiosus* act alone on the repair of damaged bases (Ishino et al., 2015). Interestingly, two incisions on either side of the damaged base by EndoQ and EndoV would generate a short hypoxanthine-containing ssDNA fragment, which could subsequently be repaired by a DNA helicase, a DNA polymerase, and a DNA ligase (Shiraishi et al., 2015). However, Ishino et al. (2015) suggested that EndoV and EndoQ are not involved in the same DNA repair pathway, which leaves open the question of how the AER pathway might work in HA.

Recently, our lab characterized the NucS endonuclease from the radioresistant hyperthermophilic euryarchaeon *Thermococcus gammatolerans* (Tga NucS). In addition to previously described activities of G and T mismatched DNA repair, we found that the Tga NucS can also recognize and cleave DNA with deaminated bases (uracil and hypoxanthine; Figure 1C; Zhang et al., 2020). Despite a very close proximity with Tko EndoMS (86% similarity), Tga NucS displays very divergent capabilities. Firstly, Tga NucS can cleave U- and I-containing dsDNA on both strands to produce a double strand break with a 4-nt 5'-overhang (Figure 1C), rather than a 5-nt 5'-overhang as observed for Tko EndoMS (Ishino et al., 2016). Both Tko EndoMS and Tga NucS can cleave hypoxanthine-containing DNA with the same efficiency as G/T mismatch (Ishino et al., 2016; Zhang et al., 2020). Tga NucS is more thermoactive than Tka EndoMS with a higher activity at physiological temperature, e.g., 80°C (Zhang et al., 2020), when Tko EndoMS works optimally at 55°C (Ishino et al., 2016). Taken together, it is clear that the Tga NucS exhibits several distinct biochemical properties from canonical NucS/EndoMS, which lead us to propose that Tga NucS might be involved in a novel alternative pathway for the repair of deaminated bases (uracil and hypoxanthine), in addition to BER and AER.

## Structure and Function of Archaeal NucS Endonucleases

Genomic analyses revealed that NucS endonucleases are widely distributed in Euryarchaea, Crenarchaea, and bacteria. Alignments of the amino acid sequences of NucS endonucleases from Archaea and bacteria showed that they possess eight highly conserved motifs, comprising mostly negatively and positively charged amino acid residues (Figure 2A). In addition, NucS endonucleases from HA harbor have a conserved QLxxLF motif, which is known to be necessary for the interaction with proliferating cell nuclear antigen (PCNA), a clamp protein as a DNA polymerase processivity (Creze et al., 2012; Ishino et al., 2018). Pab NucS interacts with Pab PCNA and the latter can modulate the activity of the former (Creze et al., 2012). Similar effects were observed in *T. kodakarensis* (Grasso and Tell, 2014; Ishino et al., 2018).

The structure of the EndoMS/NucS proteins from *T. kodakarensis* and *P. abyssi* have been solved recently (Figures 2B,C; Ren et al., 2009; Nakae et al., 2016). The data show that Tko EndoMS and Pab NucS share similar overall structures, possessing 13 β-sheet and 5 α-helix structures (Figures 2B,C; Ren et al., 2009; Nakae et al., 2016), however, the location and length of these β-sheet and α-helix structures differ between the two endonucleases. The ssDNA-binding sites for the flapped-DNA substrate (Model I) for the Pab NucS are the conserved residues W75 and Y39, which might forge stacking interactions with DNA. The ssDNA-binding sites of the splayed-DNA substrate (Model II) are residues D160, E174, K176, K44, E127, Q187, and Y191, which is a conserved motif also present in recB (Aravind et al., 2000). Mutational analysis showed that residue Y191 is a key active site residue, and residues R42, R70, and W75 are DNA binding residues of Pab NucS (Ren et al., 2009). In contrast, the key residues for mismatch base binding of the Tko EndoMS in complex with mismatched dsDNA are Y41, N76, and W77 and the catalytic activity sites are D165, E179, and K181 (Nakae et al., 2016; Ishino et al., 2018). Mutation at position D163 in the Tga NucS, which is analogous to the D165 residue of the Tko EndoMS, leads to a mutant partially lacking its uracil-containing dsDNA cleaving activity, suggesting that these residues, is also one of the catalytic activity sites in Tga NucS (Zhang et al., 2020).

## Conclusions and Future Directions

NucS endonucleases from HA are multifunctional enzymes that can cleave fork-shaped DNA, mismatched DNA, and deaminated DNA, suggesting that they can participate in several DNA repair pathways. Thus, although HA lack MutS/MutL homologues, a complete MMR system or the recognition proteins of the NER system, the NucS endonucleases, which can cleave mismatched and branched DNA, could confer HA alternatives to complete their MMR and NER pathways but also their repair pathway of deaminated bases in DNA.

However, these alternative DNA repair pathways mediated NucS endonucleases in HA still remain to be fully elucidated. Firstly, DSBs created by cleaving mismatched and deaminated DNA by the Tko EndoMS and Tga NucS appear to be a dangerous strategy for a cell, more so if under harsh environmental conditions, unless cellular HR is very effective. This is possibly the case in *Thermococcales* to which *P. abyssi*, *T. kodakarensis*, and *T. gammatolerans* belong since they are highly meroploid (Spaans et al., 2015) and have therefore multiple copies of their genomes available for DNA repair by HR (Hildenbrand et al., 2011).

Furthermore, genomic analyses have showed that in HA the *radA* gene, which codes for RadA that mediate HR and the *nucS* gene share the same promoter, suggesting that a DSB produced by the archaeal NucS endonuclease might be taken over immediately by the RadA protein. However, whether the archaeal RadA protein interacts with the archaeal NucS endonuclease to complete the endonuclease-generated DSB repair remains to be tested.

The role of the archaeal NucS endonucleases *in vivo* in cleavage of mismatched, deaminated DNA, and branched DNA remains to be clarified. Pull-down experiments will be performed by using the free archaeal NucS endonucleases or the NucS endonuclease-DNA (mismatched, deaminated, and branched DNA) as the baits to target proteins/enzymes that can interact with NucS endonucleases, which would allow to describe the complete DNA repair triggered by these archaeal NucS endonucleases.

Although several mutants of archaeal NucS endonucleases have been characterized and the crystal structures of two NucS proteins (Tko EndoMS and Pab NucS) have been solved, the detailed catalytic mechanism of the archaeal NucS endonucleases remains elusive. Furthermore, the fine characterization of additional mutants is required to provide insights into the molecular mechanism of NucS endonucleases activity since the key residues of the two enzymes differ so strongly. Finally, solving the structures of the complexes formed by archaeal NucS endonucleases with their deaminated and branched DNA substrates will be helpful to reveal structural and functional relationships of these endonucleases.

Last, both Tko EndoMS and Tga NucS, which are thermostable endonucleases, can cleave dsDNA in a manner similar to type II restriction endonucleases, suggesting that these endonucleases might be potentially used in the future for genetic engineering.

## Author Contributions

All authors listed have made a substantial, direct and intellectual contribution to the work, and approved it for publication.
